# Community health workers improve diabetes care in remote Australian Indigenous communities: results of a pragmatic cluster randomized controlled trial

**DOI:** 10.1186/s12913-015-0695-5

**Published:** 2015-02-19

**Authors:** Robyn A McDermott, Barbara Schmidt, Cilla Preece, Vickie Owens, Sean Taylor, Ming Li, Adrian Esterman

**Affiliations:** University of South Australia, 101 Currie St, Adelaide, SA 5001 Australia; James Cook University, 1 James Cook Drive, Townsville City, QLD 4811 Australia

**Keywords:** Type 2 diabetes, Australian aboriginal adults, Primary health care, Indigenous health workers

## Abstract

**Background:**

Health outcomes for Indigenous Australians with diabetes in remote areas remain poor, including high rates of avoidable complications which could be reduced with better primary level care. We aimed to evaluate the effectiveness of a community-based health-worker led case management approach to the care of Indigenous adults with poorly controlled type 2 diabetes in primary care services in remote northern Australia.

**Methods:**

Two hundred and thirteen adults with poorly controlled diabetes (HbA1c > 8.5%) and significant comorbidities in 12 remote communities were randomly assigned by service cluster to receive chronic care co-ordination from a community-based health worker supported by a clinical outreach team, or to a waitlist control group which received usual care.

**Results:**

At baseline, mean age of participants was 47.9 years, 62.4% were female, half were Aboriginal and half identified as Torres Strait Islander, 67% had less than 12 years of education, 39% were smokers, median income was $18,200 and 47% were unemployed. Mean HbA1c was 10.7% (93 mmol/mol) and BMI 32.5. At follow-up after 18 months, HbA1c reduction was significantly greater in the intervention group (−1.0% vs −0.2%, SE (diff) = 0.2, p = 0.02). There were no significant differences between the groups for blood pressure, lipid profile, BMI or renal function. Intervention group participants were more likely to receive nutrition and dental services according to scheduled care plans. Smoking rates were unchanged.

**Conclusions:**

A culturally safe, community level health-worker led model of diabetes care for high risk patients can be effective in improving diabetes control in remote Indigenous Australian communities where there is poor access to mainstream services. This approach can be effective in other remote settings, but requires longer term evaluation to capture accrued benefits.

**Trial registration:**

ANZCTR 12610000812099, Registered 29 September 2010.

## Background

Indigenous Australians have the highest prevalence and incidence of diabetes in Australia [1] and also suffer high rates of preventable complications [2]. Many of these complications can be prevented with better primary care level management however access to culturally appropriate high quality diabetes care is not always evident, especially in remote settings where there is high turnover of health staff. Australian Indigenous adults with type 2 diabetes are on average 10 years younger, have poor glycemic control and lower levels of preventive service uptake compared to non-Indigenous adults with diabetes in a national sample [3]. As a consequence there are high rates of diabetes-related avoidable hospitalisations for people in remote settings [4]. Previous reports suggest that community health workers can contribute to improved diabetes care and outcomes in high risk and under-served patients in Australia [5,6] and elsewhere through more effective communication and culturally appropriate self-management support, although until recently, few studies use robust randomized controlled designs. Interventions with the strongest outcomes included “cultural tailoring of the intervention, community educators or lay people leading the intervention, one-on-one interventions with individualized assessment and reassessment, incorporating treatment algorithms, focusing on behavior-related tasks, providing feedback, and high-intensity interventions (>10 contact times) delivered over a long duration (≥6 months)” [7].

We report the results of a cluster randomised controlled trial, “Getting better at chronic care” which aimed to evaluate the impact of a case management approach by local community-based health workers supported by an Indigenous clinical outreach team in 12 primary care services in remote far north Queensland communities over an 18 month period from 2011 to 2013.

## Methods

### Study design

The study setting was 12 small remote communities (Indigenous population range 260–3,000) in far north Queensland where the majority of the population was Aboriginal or Torres Strait Islander, served by a single provider and where the health service had agreed to participate in the trial. Primary health care is provided by either a community-controlled service (n = 4) or the Queensland Government (n = 8). The distance to the nearest tertiary hospital is between one and 12 hours by road or air.

The unit of randomisation was the community health service which was allocated to either the health-worker led case management intervention or to a waitlist control group (where the intervention was provided after 18 months). Following patient recruitment and baseline data collection, the 12 services were randomly allocated (names out of a hat) to either the intervention (n = 6) or waitlist group (n = 6). The study was not blinded as the allocation arm was known following recruitment and baseline data collection and the study was designed as a pragmatic trial reflecting effectiveness in real world practice [8]. The data reported here were collected at two timepoints. Baseline data at the time of recruitment (2011) and follow-up data in 2013.

The study was approved by the Cairns and Hinterland Institutional Ethics Committee with support from the peak Aboriginal and Torres Strait Islander Health Councils. The trial is registered as a clinical trial, ANZCTR number 12610000812099.

### Study sample

Patient eligibility criteria included having type 2 diabetes and at least one major comorbidity, age 18 or more, poor glycemic control (HbA1c > =8.5% or 69 mmol/mol), mentally competent and able to provide informed consent, and obtaining regular care from the identified health service. Exclusion criteria were major mental ill health (major psychosis or depression requiring inpatient treatment) or substance misuse, renal dialysis or end-stage renal disease, a cancer diagnosis or current pregnancy. Eligible patients were identified from their records by the health service staff, who then approached them to be in the trial. Enrolment occurred between December 2011 and July 2012. Baseline (2011) and follow-up (2013) interviews were conducted by Indigenous researchers in either plain English or Creole, depending on the preference of the patients.

**Figure 1:**
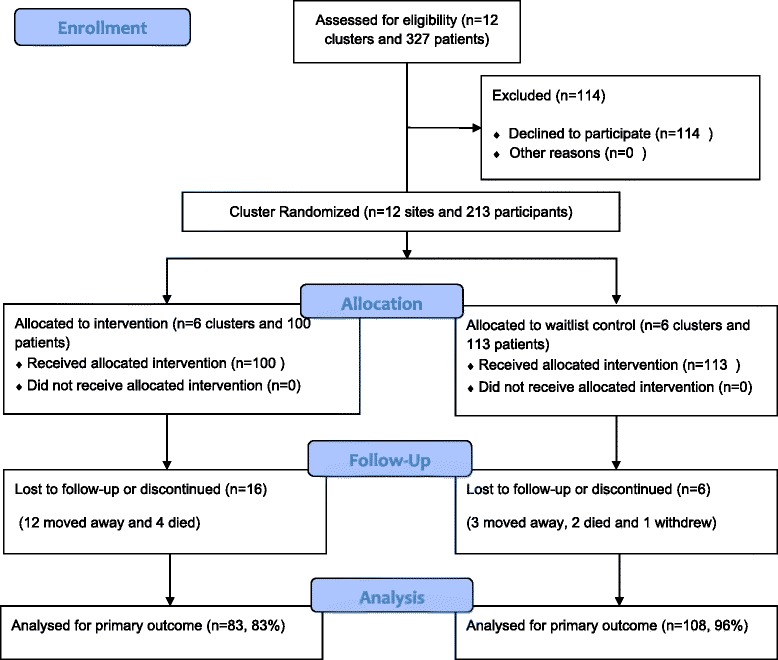
**CONSORT flow diagram: getting better at chronic care cluster RCT.**

The trial was powered to demonstrate a reduction in median HbA1c by 1.0% over 18 months compared to baseline, as the primary outcome measure. This estimate was based on a mean drop of 1.3% HbA1c over one year (from 9.9% to 8.6%, following initiation of intensive drug treatment in T2DM) reported by a large US Health Maintenance Organization [9]. A sample size of 49 in each group would have 90% power to detect a difference in mean HbA1c between the intervention and control group after 18 months of 1.0%, assuming that the common standard deviation was 1.5% using a two group *t*-test with a 0.05 two-sided significance level. With 12 communities (6 intervention, 6 control), this would require 9 subjects per community. With adjustment for clustering, assuming a design effect of 1.2 derived from a similar study [10], the required number of subjects per community is 11 for the primary outcome, or 132 in total [11]. However, due to potential difficulty of maintaining subjects in these communities in the trial, high rates of mobility, and the potential for a more modest effect size in this group, we aimed to recruit 300 subjects. In the event, 327 patients were assessed by the health services as eligible and 213 (65%) agreed to participate, providing written informed consent. Over the study period, 22 patients (10%) were lost to follow-up: 6 died, 15 moved away from the community permanently and one withdrew. More patients in the intervention group than the waitlist group were lost to follow up (Figure 1).

### Intervention

Each site allocated to the intervention arm recruited an Indigenous health worker resident in the community (selected by the health service) to work as part of the primary care team, and allocated a caseload of between 9 and 26 clients. The health workers with low caseloads worked part-time. All health workers at the commencement of the study received an intensive 3-week training in clinical aspects of diabetes and other chronic condition care, including how to support patients in self-management skills, advice on medications, routine foot care, nutrition, smoking cessation, follow up referrals to other providers, and scheduled tests. The roles of the health workers included helping patients make and keep appointments, understand their medications and nutrition and the effects of smoking and where appropriate, work with the family to help support the patient in self-management. Home visits and out-of-clinic care were features of the trial, however visits were conducted according to the patients’ preferences.

The curriculum included specific training and practice in:

- Rationale for the chronic care model and evidence-based management and treatment goals in diabetes, hypertension, COPD, renal disease and CHD.
- “Hands-on” case management: regular client home visits, including basic diabetes care (scheduled clinical checks and blood tests, counselling and referral as per the clinical guidelines supported by the clinical team).
- Working in a primary care team, with clear roles and responsibilities of team members.
- Engaging with families and using local resources to support effective client self-management.

During the 18 month intervention period, the health workers attended two workshops where they underwent refresher training, including in Good Clinical Practice and reflective practice. During these sessions, they reported on their patients’ progress and shared approaches to problem solving with the clinical support team and peers.

### Measures

The primary outcome measure for this trial, glycemic control (HbA1c) was measured by Queensland Medical Laboratories using standard high-pressure liquid chromatography methods. Blood pressure, height, weight, serum fasting lipids (Cholesterol fractions and triglycerides) were abstracted from clinic files and electronic records. Taking insulin was defined as having any of long-acting, medium- or short-acting insulin. Albuminuria was defined as urinary ACR > =3.4 mg/mmol. Estimated glomerular filtration rate (eGFR) was calculated from serum creatinine using the CKD-EPI formula, where GFR = 141 × min (Scr/κ,1) ^α^ × max (Scr/κ,1)^-1.209^ × 0.993^Age^ × 1.018 [if female] × 1.159 [if black], where Scr is serum creatinine (mg/dL), κ is 0.7 for females and 0.9 for males, α is −0.329 for females and −0.411 for males, min indicates the minimum of Scr/κ or 1, and max indicates the maximum of Scr/κ or 1 [12].

Test of Functional Health Literacy for Adults (TOFHLA) [11] was administered at study enrolment to all participants to gauge the patients’ general understanding of health messages and procedures. In general, TOFHLA was scored highly in both groups, and it was concluded that they would not have major difficulty working with the diabetes care team.

Quality of Life was estimated using the Assessment of Quality of Life (AQoL) instrument, a multi-attribute utility instrument developed using Australian importance weights [13]. Socio-demographic data was by self-report, including years of formal education, household income, employment, food insecurity (“Do you frequently not have enough money to buy food?”), current smoking and medication adherence.

Guideline recommended clinical checks, including General Practitioner Management Plans (GPMP) and specialist referrals [14], were abstracted from primary care records for the 18 months prior to the baseline and follow-up assessments. For patients with clinical indications, appropriate medication use (as indicated in the clinical guidelines) was recorded, including insulin, statins, ACEi or ARB drugs, vaccination.

Implementation Fidelity and “dose” was estimated through diaries kept by the IHWs which recorded time spent on specific intervention-related tasks versus other clinical work, as we anticipated that due to the small size of the communities and the limited number of health staff, that the trial workers would be called upon to perform regular acute clinical care to the general patient population.

### Statistical analysis

Baseline demographic and clinical data were analysed for differences between intervention and waitlist patients using t-tests, Wilcoxon rank sum tests and chi-square tests as appropriate. At follow-up all analyses were on an intention to treat basis. The 22 people who had either died or were lost to follow up were included in the dataset; however, their 18 month follow-up results were left blank. Analysis was by Generalized Estimating Equations (GEE), using a Gaussian family and identity link function. The GEE regression models used Group, Time, and a Group × Time interaction term as predictor variables. The formal test of an intervention effect was whether or not the coefficient for the interaction term was statistically significant at the 5% level. An initial analysis using a mixed effects model showed that adjustment for clustering by community made little difference, based on a likelihood ratio test. That being the case, further models were not adjusted for clustering. There were a few cases where data was available for only one time point. Where there was missing data, a second analysis was performed used multiple imputation with 20 copies of the dataset and using a regression approach for imputation. Nonparametric tests (ranksum test) were used to examine HbA1c change by GPMP exposure between groups. A further analysis was done looking at the impact of the service model (Community controlled versus Government provided) on the likelihood of clinical change, independently of group allocation.

All analyses were done using Stata version 13.

## Results

At baseline, there were no significant differences between allocation groups in age (mean age 47.9 years), sex ratio (62% women), employment status, years of schooling, median household income, self-reported food insecurity, household size, median AQoL score on the mental health scale, smoking prevalence, HbA1c (10.7%) and mean BMI (32.5). The intervention group scored lower on the health literacy test (Table 1).

**Table 1:** **Baseline socio-demographic characteristics of study participants (SD or %)**

	**Control (95% CI)**	**Intervention (95% CI)**	**All (95%CI)**	**p-value**
Number of participants	113	100	213	
Mean age (years)	47.8 (46.2-49.5)	47.9 (45.8-50.0)	47.9 (46.6-49.2)	0.948
Number (%) women	66.4 (57.6-75.2)	58.0 (48.2-67.8)	62.4 (55.9-69.0)	0.208
Unemployed (%)	52.2 (42.9-61.5)	40.0 (30.3-49.7)	46.5 (39.7-53.2)	0.204
Did not complete 12 years education (%)	61.9 (52.9-71.0)	73.0 (64.2-81.8)	67.1 (60.8-73.5)	0.344
Median annual (IQR) household income ($)	17420 (12480–33800)	20215 (13585–31200)	18200 (13000–32500)	0.598
“Not enough money for food” (%)	40.7 (31.6-49.9)	37.0 (27.4-46.6)	39.0 (32.4-45.6)	0.580
Median score (IQR) TOFLA	90.0 (81.1-94.1)	80.6 (64.9-89.0)	86.1 (71.5-92.1)	<0.001
No of people per household median (IQR)	5 (3–7)	4 (3–7)	4 (3–7)	0.608
Median AQoL mental health score (IQR) max = 1	0.93 (0.89-0.98)	0.93 (0.91-0.94)	0.93 (0.89-0.95)	0.688
Current smoker (%)	37.6 (28.4-46.8)	40.2 (30.3-50.1)	38.8 (32.1-45.5)	0.231
Mean BMI (kg/m2)*	33.0 (31.2-34.9)	31.9 (29.9-33.9)	32.5 (31.1-33.8)	0.434

*Missing data: BMI n =113.

At follow-up, 45.2% (95% CI, 34.5-56.0%) of patients in the intervention group had a current GP Management Plan (GPMP) for diabetes compared to 35.5% (26.3-44.7) in the waitlist group (OR 1.23, 95% CI, 0.72-2.22). There was no association between having a GPMP at follow-up and HbA1c change from baseline. This may be due to the fact that many GPMPs were done within 6 months of the follow-up data collection point, so the chance for the GPMP to have an immediate impact would be small. Further follow up may show a stronger relationship between having a GPMP and improved clinical indicators.

Other clinical care processes, including routine checks and specialist referrals, medications and self-reported smoking and medication adherence at baseline and follow-up, are summarised in Table 2. Intervention group patients were significantly more likely to have seen a dietician and dentist and slightly more likely to have seen a diabetes educator, be taking insulin and having influenza vaccination. Waitlist group patients showed greater self-reported adherence to prescribed medicines and were slightly more likely to have had an eye examination and be self-monitoring for glucose. Despite very high rates of dyslipidemia there was generally very low uptake of lipid lowering treatment in both groups, and the high smoking rates were unchanged. Appropriate management of albuminuria was high in both groups.

**Table 2:** **Clinical care process at baseline and follow up (%)**

	**Baseline**				**Endpoint (excluding 22 loss of follow up)**			
	**Control n = 113**		**Intervention n = 100**		**Control n = 107**		**Intervention n = 84**	
	**No**	**% (95% CI)**	**No**	**% (95% CI)**	**No**	**% (95% CI)**	**No**	**% (95% CI)**
GPMP for diabetes %	19	22.2 (13.5-30.9)	28	40.3 (29.2-51.4)	24	29.6 (19.5-39.7)	27	40.3 (28.4-52.2)
Foot check%	50	44.2 (35.0-53.5)	31	31.0 (21.8-40.2)	38	35.5 (26.3-44.7)	26	31.0 (20.9-41.0)
Seen by DM educator %	46	40.7 (31.6-49.9)	52	52.0 (42.1-61.9)	41	38.3 (29.0-47.6)	44	52.4 (41.6-63.2)
Seen by dietician %	22	19.5 (12.1-26.8)	30	30.0 (20.9-39.1)	21	19.6 (12.0-27.2)	37	44.0 (33.3-54.8)
Dentist check %	20	17.7 (10.6-24.8)	13	13.0 (6.3-19.7)	9	8.4 (3.1-13.7)	15	17.9 (9.6-26.5)
ECG check%	37	32.7 (24.0-41.5)	42	42.0 (32.2-51.8)	34	43.9 (34.4-53.4)	35	40.5 (29.8-51.1)
Eye check %	54	47.8 (38.5-57.1)	42	42.0 (32.2-51.8)	56	52.3 (42.8-61.9)	37	44.0 (33.3-54.8)
Smoker %	38	34.5 (25.6-43.5)	34	35.1 (25.5-44.7)	33	31.2 (22.4-40.4)	34	41.5 (30.7-52.2)
Blood glucose self-monitor %	45	40.9 (31.6-50.2)	46	46.0 (36.1-55.9)	63	59.4 (50.0-68.9)	44	52.4 (41.6-63.2)
Taking insulin%	55	48.7 (39.4-58.0)	40	40.0 (30.3-49.7)	47	43.9 (34.4-53.4)	40	47.6 (36.8-58.4)
Dislipidemia %	83	73.5 (65.2-81.7)	84	84.0 (76.7-91.3)	91	85.0 (78.2-91.9)	76	90.5 (84.1-96.8)
Taking lipid lowering medicines%	91	81.3 (73.9-88.6)	77	77.0 (68.7-85.3)	87	82.1 (74.7-89.5)	62	73.8 (64.3-83.3)
Albuminuria and taking ACEi or ARB drugs	46	88.5 (79.6-97.3)	47	88.7 (80.0-97.4)	58	82.9 (73.9-91.8)	51	89.5 (81.4-97.6)
Adherent to all medicines	53	46.9 (37.6-56.2)	55	55.0 (45.1-64.9)	57	53.3 (43.7-62.8)	41	48.8 (38.0-59.6)
Had Fluvax	50	44.2 (35.0-53.5)	66	66.0 (56.6-75.4)	51	47.7 (38.1-57.2)	50	59.5 (48.9-70.2)

The proportion calculated on available records. Taking insulin including having either long-term, medium-term or short-term insulin. Albuminuria defined as ACR > =3.4 mg/mmol; taking lipid lowering medicines including statin, fibrate, and lipase inhibitors.

There was a significant decrease in HbA1c of 1% from baseline in the intervention group, from 10.8% (95 mmol/mol) to 9.8% (84 mmol/mol) compared to the waitlist group, which showed a less marked decrease of 0.2% from 10.6% (92 mmol/mol) to 10.3% (89 mmol/mol), (p = 0.018). More in the intervention group achieved at least a 0.5% interval reduction in HbA1c (67.5%, 57.3-77.7), than in the waitlist group (48.6%, 38.9-58.2). There were small improvements in both groups for total cholesterol, LDL cholesterol, cholesterol: HDL ratio, with slightly better results in the intervention group. Blood pressure and weight decreased in the waitlist group and increased slightly in the intervention group. None of these effects were statistically significant at the 5% level (Tables 3 and 4).

**Table 3:** **Clinical measures at baseline and follow-up by group, absolute values**

**Pathology tests**	**Baseline**						**Endpoint (excluding 22 lost to follow up)**					
	**Control n = 113**			**Intervention n = 100**			**Control n = 107**			**Intervention n = 84**		
	**No.**	**Mean**	**SD**	**No.**	**Mean**	**SD**	**No.**	**Mean**	**SD**	**No.**	**Mean**	**SD**
HbA1c	113	10.6	1.87	99	10.8	2.0	105	10.3	2.2	84	9.8	2.3
s.creatinine	97	77.5	43.8	88	77.7	39.7	102	92.0	85.0	83	107.4	138.6
eGFR*	97	109.8	26.7	88	114.1	31.0	102	104.5	30.8	83	103.3	33.4
UACR	78	63.9	138.9	73	71.3	169.4	96	102.8	233.9	79	92.1	167.9
Cholesterol	87	4.6	1.3	81	4.5	1.3	100	4.7	1.3	79	4.4	1.4
Trig	86	2.5	1.9	81	2.1	1.4	100	2.7	1.8	79	2.5	1.8
HDL	72	1.1	0.6	79	0.9	0.2	99	0.9	0.2	78	0.9	0.2
LDL	65	2.6	1.0	76	2.7	1.1	95	2.6	1.1	71	2.4	0.9
Chol-HDL ratio	72	5.2	1.6	77	5.4	1.6	99	5.0	1.4	77	5.0	1.7
Weight	89	91.4	19.3	87	89.7	22.6	92	87.4	18.6	81	91.0	23.1
BP systolic	109	134.0	20.9	95	127.9	16.7	100	133.6	19.4	84	132.5	17.7
BP diastolic	104	81.0	11.1	92	77.7	10.4	103	81.3	11.4	84	77.8	9.9

*Calculated from serum creatinine using CKD-EPI formula.

**Table 4:** **Differences between baseline and follow-up by group**

**Measure**	**Group**	**No.**	**Mean difference**	**Std. error of the difference**	**95% CI mean** ***lower***	**95% CI mean** ***upper***
HbA1c	Control	105	−0.2	0.2	−0.7	0.2
	Intervention	83	−1.0	0.2	−1.4	−0.5
S Creatinine	Control	89	18.9	6.9	5.2	32.6
	Intervention	74	32.7	13.4	6.0	59.5
eGFR*	Control	89	−7.2	1.8	−10.7	−3.7
	Intervention	74	−10.3	3.1	−16.5	−4.1
UACR	Control	71	41.2	25.3	−9.3	91.8
	Intervention	58	17.6	20.1	−22.7	57.9
Cholesterol	Control	64	−0.3	0.2	−0.6	−0.02
	Intervention	60	−0.6	0.2	−0.9	−0.3
Triglycerides	Control	79	0.1	0.2	−0.4	0.5
	Intervention	64	0.2	0.2	−0.1	0.5
HDL	Control	64	0.06	0.02	0.02	0.09
	Intervention	62	0.06	0.02	0.02	0.1
LDL	Control	56	−0.1	0.1	−0.3	0.2
	Intervention	57	−0.3	0.1	−0.6	−0.1
Cholesterol-HDL ratio	Control	64	−0.3	0.2	−0.6	−0.02
	Intervention	60	−0.6	0.2	−0.9	−0.3
Weight	Control	72	−1.5	0.6	−2.7	−0.3
	Intervention	71	−0.6	0.7	−2.0	0.8

*Calculated using CKD-EPI formula.

Values in this table calculated as Endpoint- Baseline.

Please note, this calculation is based on only those patients for whom data was collected at both time points excluding 22 loss of follow up. There are a few cases where data was available for only one time point.

Implementation “dose” (time reported by the Health Workers spent on project-specific tasks) ranged from 43% to 78% over the intervention period. Thus we expected some diminution of effect in intervention sites.

The impact of Health Service Model (community controlled (CC) versus not CC) on the likelihood of a participant having a GPMP was explored independently of whether the trial site was in the intervention or the control group allocation. The percentage of GPMPs completed in sites with a community-controlled service was 71.0% compared to 23.5% among the non-CC sites (OR = 3.0, 95% confidence interval, 1.2-7.5 after adjustment for clustering) (Table 5). However there were no differences in clinical measures between CC and non-CC sites at follow-up.

**Table 5:** **Presence of a GPMP at T3 by health service model, independent of trial group allocation**

**GPMP**	**CC n = 62**		**Not-CC n = 151**	
	**No.**	**% (95% CI)**	**No.**	**% (95% CI)**
No	18	29.0 (17.6-40.5)	114	76.5 (69.6-83.4)
Yes	44	71.0 (59.5-82.4)	35	23.5 (16.6-30.4)

## Discussion

Type 2 diabetes and associated cardiovascular and renal disease contribute more than 60% to the 11–13 year life expectancy gap experienced by Indigenous Australians [15]. We found that a health-worker led case management approach to care of high risk adults with type 2 diabetes in remote communities in Australia was effective in improving some diabetes care processes and glycemia over 18 months. This model has been demonstrated recently in other countries and similarly disadvantaged and high risk populations [16-19] using robust evaluations, and suggest our results are generalizable to other similar rural or remote settings. However the relatively short-term (<2 years) follow-up in these studies does not allow capture of the longer term benefits. Economic evaluation therefore relies on modelling rather than empirical estimates of costs and benefits, although most reports favour low-cost primary care level interventions [20].

Limitations to our study include lower than expected patient recruitment, small numbers in two of the intervention sites, a relatively high loss to follow up (10%) which was higher in the intervention group and missing data for some of the secondary clinical endpoints. The latter was due to the pragmatic nature of the trial where clinical data was extracted from patient records. Loss to follow-up in the intervention group did not appear to be related to the trial itself, but to family circumstances, where a greater proportion moved out of the community. Other factors which potentially limited the implementation effectiveness of the study were major health system reform occurring in the Queensland government health services generally during the life of the trial, which limited the ability of the service to recruit and retain essential staff. These changes disproportionately affected 3 of the intervention sites. Process evaluation found that all six health workers experienced higher workloads as the services pressured them to undertake clinical work in addition to their study caseload. This tended to dilute the potential impact of their work on the care of study patients.

## Conclusions

In summary, we have demonstrated a significant and favourable impact on some diabetes care processes and glycemic control of a community health worker-led model of diabetes care in high risk disadvantaged populations in remote Australia. A longer term evaluation may enable more complete capture of further benefits, including economic impact on the health service and patient-important outcomes. As the rising incidence of obesity, diabetes and associated complications rises globally, it is clear that the current health care workforce, especially in low- and middle-income countries will not be able to adequately manage new and established cases using current workforce models [21]. Community Health Workers in high income countries have been shown to contribute significantly to chronic disease management especially in hard-to-reach populations [22], although urban settings might offer different challenges [23] and may play a more important role in the primary care team as the diabetes epidemic increases in these groups.

## Abbreviations

ACEi: Angiotensin converting enzyme inhibitor
ACR: Albumin:creatinine ratio
ARB: Angiotensin receptor blocker
ANZCTR: Australian and New Zealand Clinical Trials Register
AQoL: Assessment of Quality of Life
CC: Community controlled
CHW: Community health worker
COPD: Chronic obstructive pulmonary disease
CHD: Coronary heart disease
GEE: Generalized estimating equations
GPMP: General practitioner management plan
HbA1c: Haemoglobin A1c
HDL: High density lipoprotein cholesterol
T2DM: Type 2 diabetes mellitus
TOFHLA: Test of functional health literacy for adults
